# Broadband Plasmon-Enhanced Four-Wave Mixing in Monolayer MoS_2_

**DOI:** 10.1021/acs.nanolett.1c02381

**Published:** 2021-07-19

**Authors:** Yunyun Dai, Yadong Wang, Susobhan Das, Shisheng Li, Hui Xue, Ahmadi Mohsen, Zhipei Sun

**Affiliations:** † Department of Electronics and Nanoengineering, Aalto University, Espoo 02150, Finland; ‡ International Center for Young Scientists (ICYS), National Institute for Materials Science (NIMS), Tsukuba 305-0044, Japan; § QTF Centre of Excellence, Department of Applied Physics, Aalto University, Espoo 02150, Finland

**Keywords:** Two-dimensional materials, nonlinear optics, four-wave mixing, plasmonic enhancement, MoS_2_

## Abstract

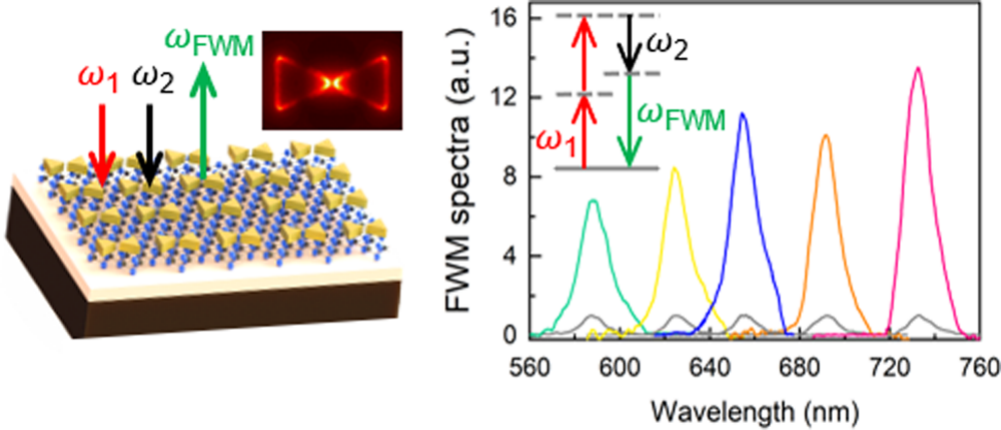

Two-dimensional transition-metal dichalcogenide monolayers have remarkably large optical nonlinearity. However, the nonlinear optical conversion efficiency in monolayer transition-metal dichalcogenides is typically low due to small light–matter interaction length at the atomic thickness, which significantly obstructs their applications. Here, for the first time, we report broadband (up to ∼150 nm) enhancement of optical nonlinearity in monolayer MoS_2_ with plasmonic structures. Substantial enhancement of four-wave mixing is demonstrated with the enhancement factor up to three orders of magnitude for broadband frequency conversion, covering the major visible spectral region. The equivalent third-order nonlinearity of the hybrid MoS_2_-plasmonic structure is in the order of 10^–17^ m^2^/V^2^, far superior (∼10–100-times larger) to the widely used conventional bulk materials (e.g., LiNbO_3_, BBO) and nanomaterials (e.g., gold nanofilms). Such a considerable and broadband enhancement arises from the strongly confined electric field in the plasmonic structure, promising for numerous nonlinear photonic applications of two-dimensional materials.

Nonlinear optics in the nanoscale regime has attracted massive attention in the last decades.^1^ For example, it provides a host of fascinating phenomena (e.g., saturable absorption),^2^ which are remarkably useful for photonic applications such as ultrafast pulse generation.^3−5^ Among various nonlinear optical processes, four-wave mixing (FWM), a third-order optical nonlinear process, plays a key role for a large range of applications (such as frequency conversion, signal amplification, and optical switching).^6,7^ Recently, two-dimensional (2D) transition-metal dichalcogenides (TMDs)^8^ have attracted tremendous interest due to their unique physical properties, such as strong excitonic effect,^9,10^ dynamic electrical tunability,^11,12^ and large optical nonlinearity.^13−22^ As newly emerging nanoscale nonlinear materials, TMDs exhibit fascinating optical nonlinearity such as the excitonic enhancement of harmonic generation.^19,23^ Especially, recently the gate-tunable second-harmonic generation (SHG)^24^ and FWM^25^ have been realized in TMDs.^26^ All these results show the great potential of using TMDs for diverse on-chip nonlinear optical devices, fundamentally different from those based on traditional bulk materials.^1,25^

However, the applications of TMDs in nonlinear optics are limited due to the low conversion efficiency caused by the short light–matter interaction length at their atomic thickness. Several approaches have been proposed including plasmonics,^27−30^ photonic cavities,^31^ and waveguide integration.^32^ Among them, plasmonics provides an excellent platform for enhancing light–matter interaction, which shows great potential for nanoscale nonlinear optical applications.^33−37^ For example, the SHG of WS_2_ on the silver nanogroove grating was enhanced by the plasmonic resonance, with a large enhancement factor (∼400).^33^ Besides, SHG of bilayer WSe_2_ was obtained by the plasmonic hot carrier injection.^38^ Nevertheless, plasmonic enhancement of FWM in TMDs has not been studied yet and deserves further investigation.

Here, for the first time, we report broadband FWM enhancement in monolayer MoS_2_ with plasmonic structures. An enhancement factor up to three orders of magnitude is achieved compared to the FWM generated from bare MoS_2_ monolayer without plasmonic structures. The massive enhancement is attributed to the strongly confined electric field of the pump light in the hot spots of the plasmonic structures. The FWM enhancement with different excitation polarization states and plasmonic structure dimensions is also investigated. Furthermore, for the first time, we demonstrate a broadband (up to ∼150 nm) enhancement of FWM in the hybrid MoS_2_-plasmonic structure with an in-depth discussion about the broadband enhancement mechanism. The plasmon-induced significant and broadband FWM enhancement in 2D materials is promising for numerous applications in the future nonlinear photonics.

## Results and Discussion

### MoS_2_-Plasmonic Nanostructures

**Figure 1:**
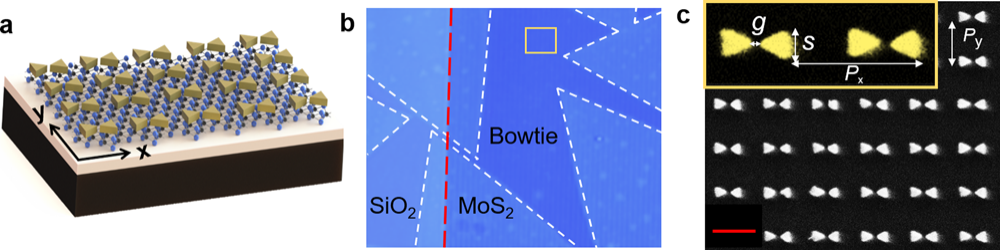
Hybrid MoS_2_-plasmonic nanostructures. (a) Schematic illustration and (b) optical image of the MoS_2_-plasmonic structure (Au bowtie) on a Si/SiO_2_ substrate. In the optical image, the white dashed lines outline the edges of the MoS_2_ flakes, and the red dashed line outlines the boundary of the plasmonic nanostructures and the bare SiO_2_/Si substrate. (c) SEM image of the Au bowtie nanostructures. Scale bar: 500 nm. Inset: zoomed image of Au bowtie nanostructures. Structure parameters are also labeled.

A schematic layout and an optical image of the hybrid MoS_2_-plasmonic structure are shown in Figure 1a and b. Monolayer MoS_2_ flakes grown on a SiO_2_/Si substrate by the chemical vapor deposition (CVD) method^39^ are of the triangular shape and appear lighter colored compared to the substrate. By examining Raman and photoluminescence spectra (Figure S1, Supporting Information), the CVD MoS_2_ flakes are identified as monolayers. The 50 nm-thick gold nanostructures are then patterned on top of the MoS_2_ flakes (fabrication details in the Supporting Information). The scanning electron microscopy (SEM) image of a typical Au bowtie array is shown in Figure 1c. The size of each equilateral triangle (*s*) is ∼160 nm, and the gap (*g*) is ∼30 nm, with the unit cell pitch *P*_*x*_ = *P*_*y*_ = ∼ 600 nm.

### Plasmon Enhanced FWM in Monolayer MoS_2_

**Figure 2:**
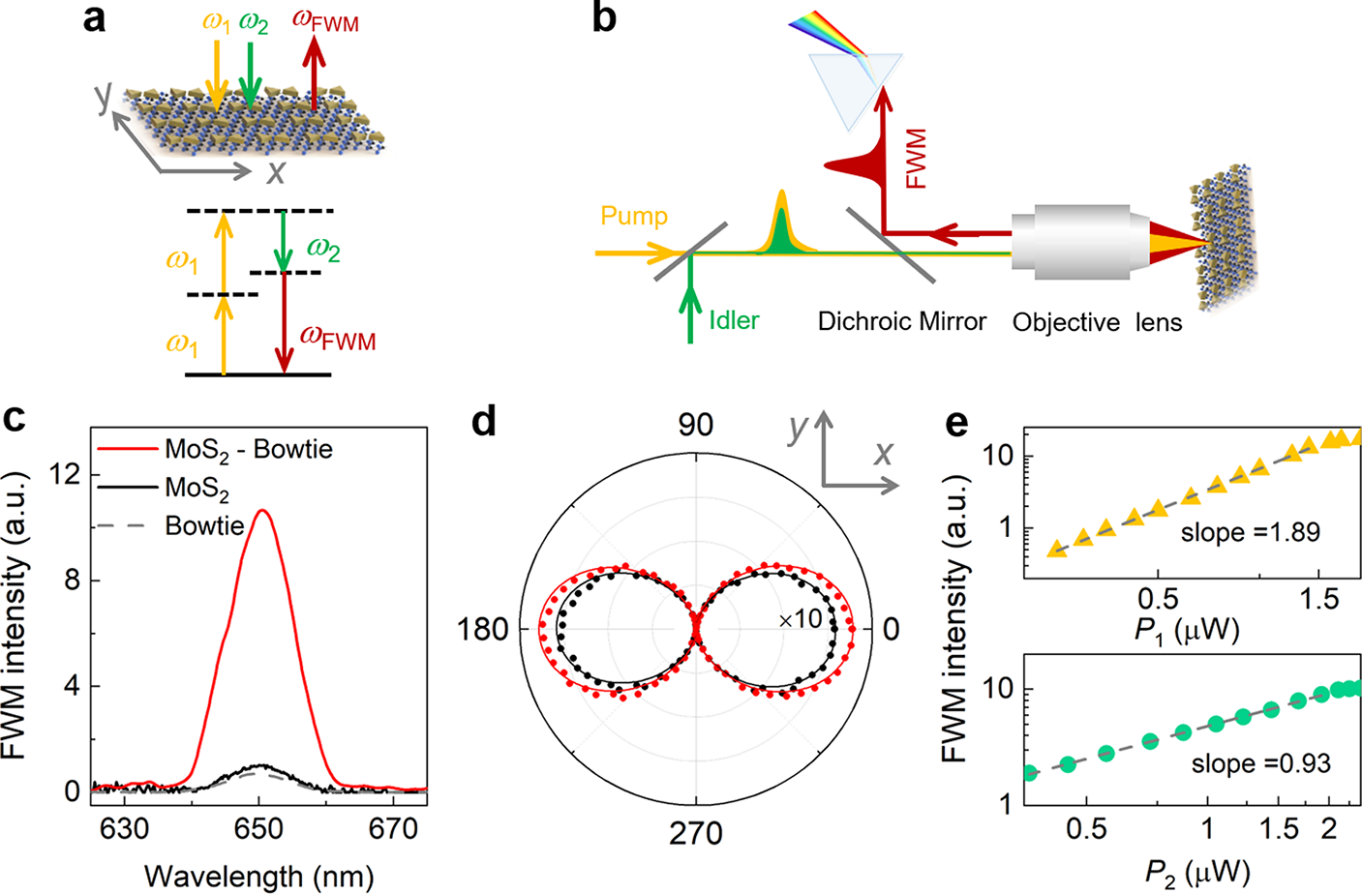
Plasmon-enhanced FWM in monolayer MoS_2_. (a) Illustration of FWM from the hybrid MoS_2_-plasmonic structures (upper panel) and the energy level diagram of FWM process (lower panel). (b) Schematic of the experimental setup for nonlinear optical measurements. (c) FWM spectra measured from hybrid MoS_2_-plasmonic structures (red curve), bare MoS_2_ monolayer without plasmonic structure (black curve), and the plasmonic structure only (gray dashed curve). (d) Generated FWM signals from the hybrid MoS_2_-plasmonic structures (red) and the bare MoS_2_ monolayer (black) through a polarization analyzer are plotted as a function of angle θ between the polarization analyzer axis and the *x*-axis. The experiment data (dots) can be well fitted by a cos^2^ θ curve. (e) Dependence of experimental FWM peak intensities on the average power of the pump (*P*_1_) and probe light (*P*_2_), with a fit to a power law *I*^m^. Upper panel: Dependence of FWM on *P*_1_ with a fit (*m* = ∼1.89). Lower panel: Dependence of FWM on *P*_2_ with a fit (*m* = ∼0.93).

An illustration of the FWM process in the hybrid MoS_2_-plasmonic structure is shown in Figure 2a. The MoS_2_ sample, excited by pump and idler beams with frequencies at ω_1_ and ω_2_ (ω_1_ > ω_2_), generates FWM signals at ω_FWM_ = 2ω_1_ – ω_2_, following the law of conservation of energy. The lower panel in Figure 2a shows the energy level diagram of the FWM process. To measure the FWM signals, a home-built femtosecond laser based microscopic setup (Figure 2b) is employed. The input pump and idler laser beams are linearly polarized with polarizations parallel along the *x*-axis. For other polarizations (e.g., the pump and idler beams are cross-polarized), they are fully discussed in Figure S3 of the Supporting Information. The pump and idler beams are spatially merged using a dichroic mirror and are temporally synchronized by a delay line. The combined beams are focused on the sample through an objective lens. The generated nonlinear optical signals in the MoS_2_ sample are measured in a reflection configuration by a spectrometer.

Here, we use a pump photon energy (ℏω_1_) at ∼1.55 eV (λ_1_ = ∼800 nm) and an idler photon energy (ℏω_2_) at ∼1.19 eV (λ_2_= ∼1040 nm). Then the generated FWM photon energy (ω_FWM_ = 2ω_1_ – ω_2_) is at ∼1.91 eV (λ_FWM_= ∼650 nm). In the experiment, the average powers for both pump and idler input light are fixed as ∼1 μW (with a corresponding peak intensity of ∼44 GW/cm^2^) unless otherwise specified. The plasmonic structures are fabricated on one portion of a few MoS_2_ monolayer flakes (Figure 1b), which allows for a self-consistent comparison of FWM from the same MoS_2_ flake with and without plasmonic structures. As shown in Figure 2c, the FWM peak intensity measured from the MoS_2_-plasmonic structures is one order of magnitude (∼11-fold) higher than that from the bare MoS_2_ region (i.e., without the plasmonic structure) and the pristine bowtie plasmonic structure. The results fully demonstrate the significant plasmonic enhancement of the third-order optical nonlinearity in 2D materials. Apart from FWM, there also exist multiple nonlinear optical processes in the hybrid MoS_2_-plasmon nanostructures. Figure S4 in the Supporting Information presents the spectra of the multiple nonlinear processes (e.g., SHG, Sum frequency generation, and FWM) on bare MoS_2_ and hybrid MoS_2_-plasmon nanostructures.

The polarization of the generated FWM signal is also measured. Here, both the pump and idler beams are linearly polarized along the *x*-axis, and a polarization analyzer for the generated FWM signal is set at an angle θ with respect to the *x*-axis. Figure 2d presents the FWM versus θ taken on bare MoS_2_ monolayer (i.e., without plasmonic structures) and the hybrid MoS_2_-plasmonic structures, respectively. The observed FWM signals from both the bare MoS_2_ and the hybrid MoS_2_-plasmonic structures are linearly polarized along the *x*-axis, fitted well by cos^2^ θ, agreeing well with the previous experimental FWM results.^22^

The power dependence of the generated FWM signal from the hybrid MoS_2_-plasmonic structures is examined by changing the pump power *P*_1_ and the idler power *P*_2_, respectively. The upper panel of Figure 2e shows the peak intensities of the FWM as a function of *P*_1_ in a log–log scale, while *P*_2_ = ∼1 μW. The lower panel of Figure 2e shows the peak intensities of FWM spectra as a function of *P*_2_ on a log–log scale, while *P*_1_ = ∼1 μW. It roughly follows a square and linear power-law behavior as a function of the pump power and idler power, respectively, which confirms the detected signal is generated by the FWM process when excited by the pump and idler beams. Note that it gets slightly saturated at high pump/idler powers possibly due to the intrinsic loss (e.g., multiphoton absorption) of the pump light and FWM signals,^40^ and the shift of the plasmonic resonance (e.g., shape deformation of the nanostructure) at high incidence power.^41^

For a quantitative comparison, the experimental enhancement factor (*EF*_ex_) for FWM in the hybrid MoS_2_-plasmonic structure is calculated from the results shown in Figure 2c. Considering that the hot spot of the plasmonic structure (i.e., the gap of the Au bowtie nanostructure) uniformly occupies a small area in the array, the *EF*_ex_ can be approximately calculated as^42^
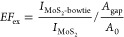
1where *I*_MoS_2_-bowtie_ and *I*_MoS_2__ are the measured FWM intensities from MoS_2_ with and without the bowtie plasmonic structure, *A*_0_ represents the area of the unit cell in this array, and *A*_gap_ represents the hot spot area of the plasmonic structure (i.e., the gap of the bowtie nanostructure). Accounting for the small hot spot area fraction (*A*_gap_/*A*_0_ = ∼0.25%) and ∼11-fold FWM enhancement () from the hybrid MoS_2_-plasmonic structures, the estimated maximum *EF*_ex_ at the plasmonic hot spot is calculated to be ∼4400 for the incident beam polarized along the *x*-axis shown in Figure 2. In addition, we also estimate the theoretical enhancement factor (*EF*_th_) of ∼5250 (details in the Supporting Information), which agrees with our experimental results. Therefore, the plasmonic resonance strongly enhances the light–matter interactions in 2D materials, and the enhancement factor up to three orders of magnitude is achieved.

### FWM Enhancement with Different Polarizations

**Figure 3:**
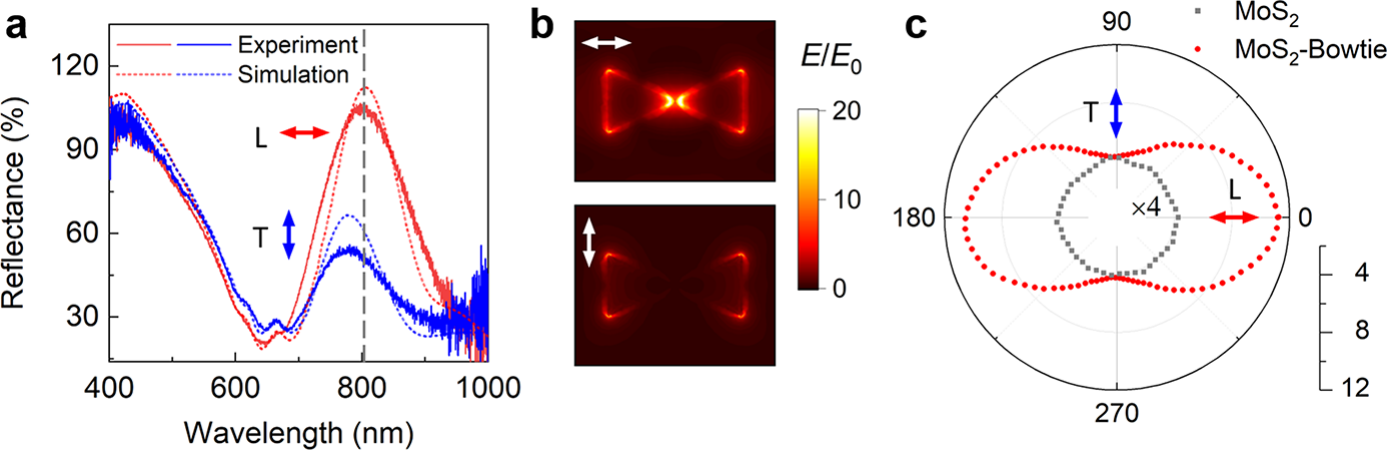
FWM polarization dependence. (a) Experimental (solid curves) and simulated (dotted curves) reflection spectra, (b) simulated electric field at 800 nm at different input polarizations (upper, the longitudinal mode; lower, the transverse mode). (c) Experimental FWM intensity from bare MoS_2_ (gray dots) and hybrid MoS_2_-plasmonic structure (red dots) as a function of polarization angle α between the polarization of incident beams and the *x*-axis.

The bowtie nanostructures typically support different plasmonic modes along with *x* and *y* directions. When the incident light is linearly polarized along the *x*-axis, the longitudinal plasmonic modes are excited. While the incident light is linearly polarized along the *y*-axis, the transverse plasmonic modes are excited. Here, we study the polarization dependence of the hybrid MoS_2_-plasmonic structure. The experimental reflection spectra of the hybrid MoS_2_-plasmonic structure on the SiO_2_/Si substrate are measured with *x* and *y* polarizations, agreeing well with the simulated reflection spectra, as shown in Figure 3a. The relative reflection spectra (*R* = *R*_MoS_2_-bowtie_/*R*_sub_) feature broad peaks for both polarizations, where *R*_MoS_2_-bowtie_ is the reflection from the hybrid MoS_2_-plasmonic structures and *R*_sub_ is the reflection from a bare SiO_2_/Si substrate. Note that the small peaks at around 620 and 660 nm are the B- and A-excitonic states of MoS_2_, clearly observed both in the experimental and simulated spectra. The significant peaks in the reflection spectra are attributed to the longitudinal and transverse plasmonic resonances, as confirmed by simulated electric field enhancement in the bowtie nanostructures at the wavelength of 800 nm (Figure 3b). The detailed simulations are introduced in Figure S5 in the Supporting Information. The longitudinal plasmonic resonance (ℏω_p_L_) is at ∼1.55 eV (λ_p_L_ = ∼800 nm), while the transverse plasmonic resonance is at a slightly higher energy ℏω_p_T_ = 1.63 eV (λ_p_T_ = ∼760 nm). For transverse mode, no interaction between the two neighboring triangular nanostructures is observed (the lower panel in Figure 3b), which corresponds to the dark spot in the gap. In contrast, for the longitudinal mode, the plasmonic modes supported by two neighboring triangular structures within one bowtie structure are strongly coupled, leading to the relative redshift of the longitudinal plasmonic mode.^43^ The hot spot inside the gap of the bowtie structure as illustrated in the upper panel of Figure 3b is an evidence for this strong interaction. We observe that the electric field enhancement with the longitudinal mode within the plasmonic structure (i.e., the gap of the bowtie) is much stronger than that of the transverse mode where the electric filed is only enhanced along the two sides of the bowtie.^44^

The longitudinal and transverse plasmonic modes have totally different resonances (e.g., resonant wavelengths, electric fields), resulting in different enhancement behaviors in the nonlinear optical process. Here, we study the angular dependence of the FWM enhancement in the hybrid MoS_2_-plasmonic structure. Figure 3c shows the experimental FWM peak intensity measured from monolayer MoS_2_ with and without the plasmonic structure as a function of the polarization angle α between the incident beams and the *x*-axis. Note that the pump and idler beams are linearly polarized with polarizations parallel with each other. The details of the measurement setup are shown in Figure S6a in the Supporting Information. In contrast to the isotropic FWM from bare MoS_2_, the FWM intensity from the hybrid MoS_2_-plasmonic structure varies with α. When the pump laser is polarized along the *x*-axis (α = 0°), it is resonant with the longitudinal plasmonic mode at the wavelength of 800 nm, the FWM enhancement reaches the maximum (∼11-fold), higher than that from the transverse mode (∼4-fold) with the *y*-axis polarized excitation (α = 90°). The spectra of the enhanced FWM signals at α = 0° and 90° are shown in Figure S6b in the Supporting Information. Between the two critical angles where is a superposition of two plasmonic modes, the enhancement of the nonlinear optical process varies between the maximum to the minimum. Therefore, tuning the polarization of the pump laser enables the modulation of the plasmons in the nanostructures, thus tuning the FWM intensity of the hybrid MoS_2_-plasmonic structure.

### FWM Enhancement with Different Plasmonic Structure Dimensions

**Figure 4:**
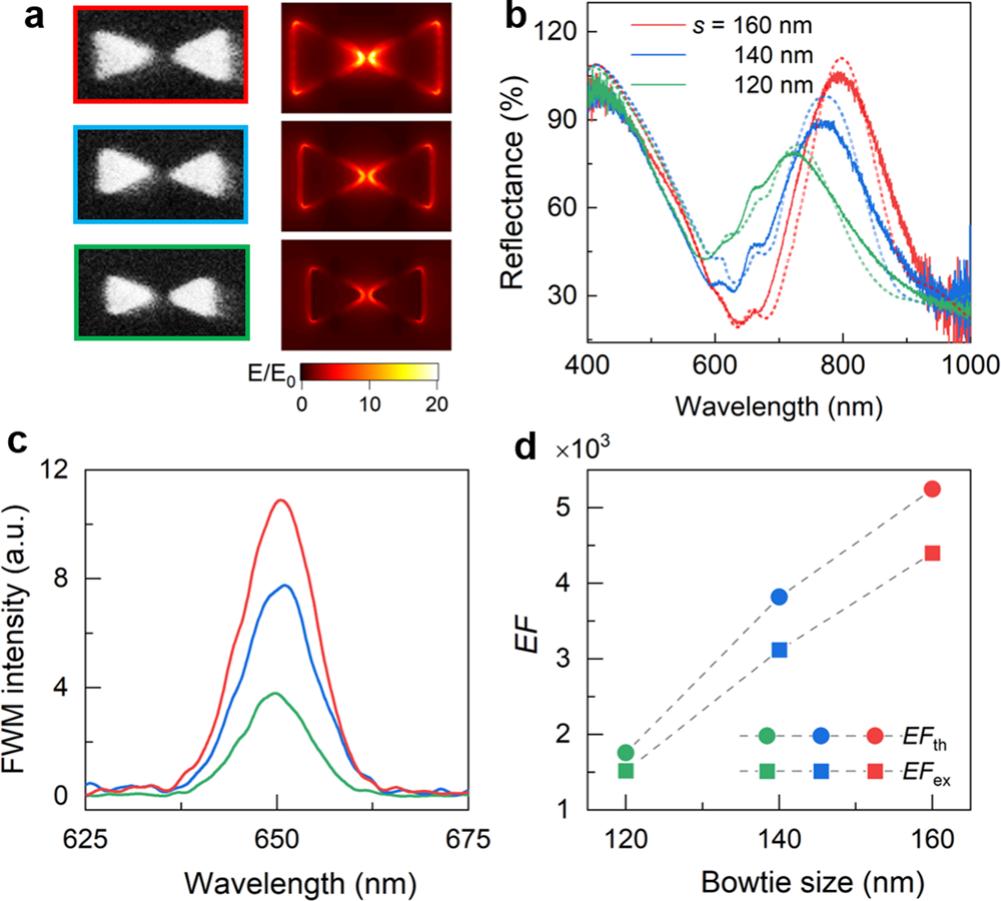
FWM enhancement with different plasmonic structure dimensions. (a) SEM images and the simulated electric field at 800 nm of Au bowtie structures with different sizes (*s* = 160, 140, and 120 nm). (b) Experimental (solid curves) and simulated (dotted curves) relative reflection spectra, (c) experimental FWM spectra, and (d) experimental (*EF*_ex_) and theoretical (*EF*_th_) enhancement factors of the corresponding MoS_2_-plasmonic structures.

To further understand the plasmon-enhanced FWM process, we fabricate Au bowtie nanostructures with different dimensions on top of monolayer MoS_2_. The SEM images of the patterned Au bowties are shown in the left panel of Figure 4a, with structure size *s* varying from 160 to 120 nm, while the gap (*g* = 30 nm) and the pitch (*P*_*x*_ = *P*_*y*_ = 600 nm) are fixed. The simulated electric field for the longitudinal polarization at 800 nm is presented in the right panel of Figure 4a. Figure 4b presents the experimental relative reflection spectra (*R* = *R*_MoS_2_–bowtie_/*R*_sub_) measured from the Au nanostructure arrays on monolayer MoS_2_ with the longitudinal polarization, agreeing well with the simulated reflection spectra. Details of the simulated reflection spectra are shown in Figure S5 in the Supporting Information. The plasmon resonance shows the redshift from 740 to 800 nm with the increment of the structure size *s*. The strengths of the plasmonic resonance, shown as the amplitude of the reflection peaks, are stronger with the increasing structure size, which fits well with the simulation results of the electric field in Figure 4a.

We further experimentally investigate the effect of the structure dimensions on the FWM enhancement in monolayer MoS_2_. The corresponding FWM spectra are measured from the hybrid MoS_2_-plasmonic structures with different nanostructure sizes (Figure 4c). Thus, the experimental (*EF*_ex_) and theoretical (*EF*_th_) enhancement factors can be calculated, respectively, as shown in Figure 4d. When nanostructure size *s* changes from 120 to 160 nm, both the experimental and theoretical enhancement factors increase and show a similar tendency (*EF*_ex_ from 1500 to 4400, and *EF*_th_ from 1750 to 5250). We note that the nanostructure with *s* = 160 nm gives the highest *EF*_ex_ with on-resonance excitation at 800 nm, while the FWM intensity drops with decreased nanostructure sizes, as expected from the simulation results (Figure 4a). Note that if the size of the nanostructure further increases (i.e., *s* > 160 nm), the plasmonic resonance is expected to redshift to a longer wavelength (i.e., >800 nm), and thus, the pump laser at 800 nm does not match the plasmonic resonance, leading to the suppression of the FWM enhancement.

### Broadband FWM Enhancement

**Figure 5:**
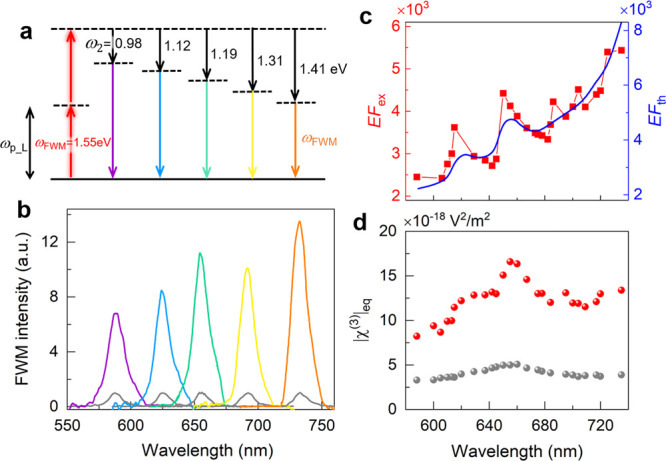
Broadband FWM enhancement. (a) Concept of the broadband plasmon-enhanced FWM, where the pump light frequency matches the plasmonic resonance and the idler light frequency changes to generate tunable FWM enhanced in a broad spectral range. (b) Experimental FWM signals at different wavelengths from the hybrid MoS_2_-plasmonic structure (colored curves) and bare MoS_2_ (gray curves). The FWM intensity from bare MoS_2_ is normalized. (c) Wavelength-dependent experimental (red dots) and theoretical (the blue curve) enhancement factors. (d) Calculated third-order equivalent nonlinear coefficient of FWM in hybrid MoS_2_-plasmonic structure (red dots) and third-order nonlinear coefficient of bare MoS_2_ (gray dots).

The schematic of broadband FWM enhancement is shown in Figure 5a. In our experiments, we fix the pump frequency and change the idler frequency to generate the tunable FWM enhancement in a broad wavelength range. In our case, since the fixed pump frequency matches the plasmonic resonance (i.e., ω_1_ = ω_p_L_), the FWM process is always kept on resonance. Hence, regardless of the tunable idler frequency, the FWM signal will be enhanced over a wide spectral range. To demonstrate the broadband FWM enhancement concept, our pump (ℏω_1_) is fixed at ∼1.55 eV (i.e., λ_1_ = ∼800 nm) on resonance with the plasmonic resonance (ω_1_ = ω_p_L_), and the idler (ℏω_2_) changes from ∼0.98 to 1.41 eV (i.e., λ_2_ = ∼1260 nm −880 nm), limited by the laser operation range. Both the pump and idler beams are linearly polarized along the *x-*axis. As a result, the generated FWM (ω_FWM_ = 2ω_1_ – ω_2_) is tunable from ∼2.12 to 1.69 eV (λ_FWM_ = ∼588–734 nm), covering the major part of the visible wavelength region. As shown in Figure 5b, the experimental FWM intensity measured from the hybrid MoS_2_-plasmonic structure is significantly higher than that from the bare MoS_2_ over the wide spectral range of 588–734 nm (∼150 nm). This result demonstrates that one single plasmonic structure can offer a broadband platform for enhancing FWM and other similar multiwave mixing processes in 2D materials, pushing the previously demonstrated limitation of a narrow spectral range of enhancement such as the cavity-enhanced SHG in 2D materials toward the broadband scheme.^31^

The wavelength-dependent experimental and theoretical enhancement factors are calculated, respectively, as shown in Figure 5c. Over the spectral range, both *EF*_ex_ and *EF*_th_ increase at longer wavelengths. It is mainly because FWM emission is enhanced when the FWM frequency and the idler frequency approach the plasmonic resonance at the wavelength of 800 nm (Figure S7, Supporting Information). Moreover, *EF*_ex_ and *EF*_th_ peaks at around 620 and 660 nm are attributed to the enhanced interaction between MoS_2_ and the plasmonic resonance at exciton wavelengths. The detailed discussion is in Figure S7 of the Supporting Information.

On the basis of the measured FWM intensities, the wavelength-dependent third-order nonlinear coefficients |χ^(3)^| of bare MoS_2_ and the equivalent nonlinear coefficient |χ^(3)^|_eq_ of the hybrid MoS_2_-plasmonic structures are calculated from ∼588 to 734 nm, as shown in Figure 5d. The detailed calculation method is discussed in the Supporting Information. Attributed to the plasmonic enhancement, the equivalent |χ^(3)^|_eq_ of MoS_2_ in the hybrid MoS_2_-plasmonic structure is in the order of 10^–17^ m^2^/V^2^, which is almost one order of magnitude larger than the previous results and far better than the conventional nonlinear optical materials (such as LiNbO_3_, BBO, Tables 1 and 2 in the Supporting Information).^1^ To further improve the FWM enhancement, we can optimize the nanostructures for improved field enhancement (e.g., shrink the periodicity with a higher filling fraction of nanostructures, narrow down the gap within the bowtie). Besides, we can integrate the hybrid MoS_2_-plasmonic structures into an optical cavity or a waveguide to improve the enhancement.

## Conclusions

To summarize, we have demonstrated the broadband enhanced nonlinear light–matter interaction in MoS_2_ with plasmonic structures. The enhancement factor of FWM up to three orders of magnitude is achieved. The enhancement is attributed to the localized electric field of the pump beam in the hot spot of the plasmonic nanostructures. With the longitudinal plasmonic mode, the plasmonic resonance with the extremely enhanced electric field at the hot spot results in the larger FWM enhancement compared to that of the transverse plasmonic mode. Moreover, a broadband FWM enhancement is realized over 150 nm in the visible spectral range. Our results show that the plasmonic structures can drastically improve the broadband nonlinear light–matter interactions in hybrid MoS_2_-plasmonic structures, boosting the applications of 2D materials for future nonlinear optical devices.

## Supporting Information Available

Device fabrication and the characterization of monolayer MoS_2_, nonlinear optical measurement, polarization dependence of FWM, full spectra of different nonlinear optical processes, numerical simulation method, polarization dependence of FWM enhancement, theoretical calculation of enhancement factors, calculation of nonlinear coefficients for FWM signals in MoS_2_, typical third-order nonlinear coefficients reported on 2D layered materials, enhancement factors for nonlinear coefficients of two-dimensional materials in hybrid structures (PDF)
