# Supplementary material for: Broadband Plasmon-Enhanced Four-Wave Mixing in Monolayer MoS2
Source: Nano Lett. 2021 Jul 19;21(14):6321–7. doi: 10.1021/acs.nanolett.1c02381 (PMC8323120; doi:10.1021/acs.nanolett.1c02381)
Supplement: Supplementary file 1 — nl1c02381_si_001.pdf [file nl1c02381_si_001.pdf]

Supporting information for

Broadband Plasmon-Enhanced Four-Wave Mixing in Monolayer MoS_2

Yunyun Dai[†,], Yadong Wang[†], Susobhan Das[†], Shisheng Li[§], Hui Xue[†], Ahmadi Mohsen[†], and Zhipei Sun[†,#,*]*

†Department of Electronics and Nanoengineering, Aalto University, Espoo 02150, Finland

§International Center for Young Scientists (ICYS), National Institute for Materials Science (NIMS), Tsukuba 305-0044, Japan

#QTF Centre of Excellence, Department of Applied Physics, Aalto University, Espoo 02150, Finland

*Email: zhipei.sun@aalto.fi;yunyun.dai@aalto.fi

1. Device fabrication and MoS_2 flake characterization

Monolayer MoS_2 is grown on a SiO_2/Si substrate by chemical vapor deposition (CVD) method. Bowtie structures are patterned on the as-grown MoS_2 substrate by electron beam lithography (Vistec 6000). A 50 nm Au film is evaporated by electron beam evaporation system and then lifted off after soaking in acetone for 4 h. The size of the plasmonic bowtie array is 50×50 μm^2.

Monolayer MoS_2 film is prepared by chemical vapor deposition (CVD) method. By examining Raman and photoluminescence spectra (Figure S1), the CVD MoS_2 flakes are identified as monolayers. Raman spectrum shows peaks at ~382 (E_{2g}) and 402 cm^{-1} (A_{1g}), with the peak distance of ~20 cm^{-1}, indicating monolayer MoS_2[1]. Further, photoluminescence (PL) spectrum is measured with two strong peaks at ~620 and 670 nm, corresponding to B and A excitons respectively, as shown in Figure S1(b) [1, 2]. The Raman and PL spectra are measured with excitation of a continuous-wave laser at 532 nm with the power of ~1 mW.

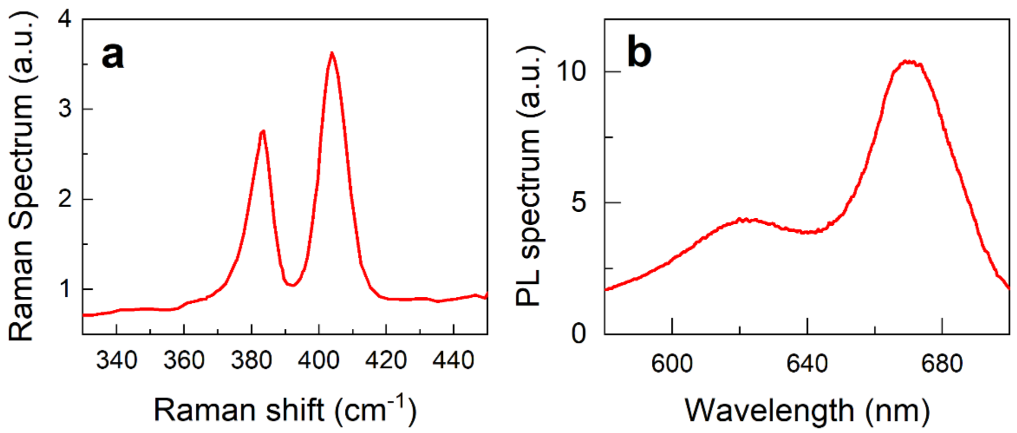

Figure S1 Raman spectrum (a) and PL spectrum (b) of CVD MoS_2 film.

2. Nonlinear optical measurement

A home-built femtosecond laser based microscopic system is employed for measuring the nonlinear optical signals from the fabricated samples. The schematic of this system is shown in Figure 2b. A femtosecond parametric amplifier (Spectra-Physics, TOPAS) is used for optical excitation with a repetition rate of 2 kHz. The pump and idler laser beams for optical excitation, are linearly polarized with polarization directions parallel with each other. The spectra of the pump and idler laser beams are shown in Figure S2. The polarization of the laser beam is changed via a half waveplate. The pulse duration of the incident pulses is ~230 fs. The pulses of the pump and idler beams are spatially merged through a dichroic mirror and temporally synchronized by a delay line, focused collinearly on the sample through an objective lens

(NA=0.75, 40×). The spot sizes of the pump and idler beams on the sample are ~2.5 μm. The FWM signal generated in the sample is detected by a detector following a monochromator (Andor 328i) in the reflection configuration.

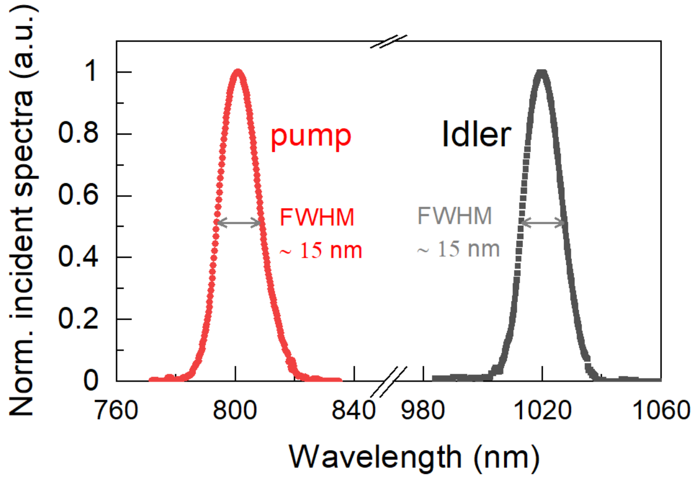

Figure S2 Normalized spectra of the pump and idler light centered at ~800 nm and ~1020 nm.

3. Polarization dependence of FWM

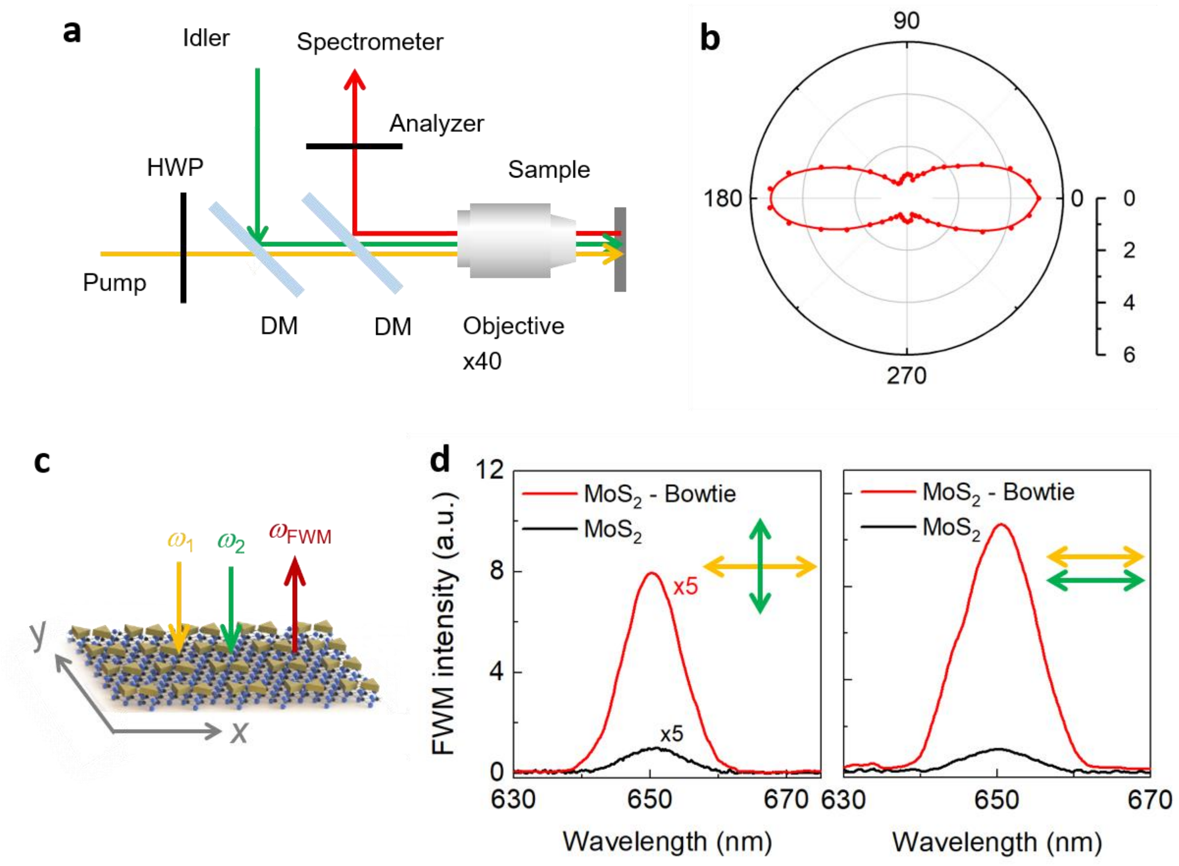

Figure S3 Polarization dependence of FWM. (a) The illustration of the setup. The idler beam is linearly polarized along *x*-axis throughout the measurement. The polarization of the pump

beam is changed via a half-wave plate (HWP). The angle between the polarization of the pump beam and the idler beam is defined as β. (b) The experimental FWM total intensity as a function of β. (c) The cross-polarization with the pump polarized along *x*-axis and probe polarized along *y*-axis on the hybrid MoS_2-plasmonic structures. (d) The FWM spectra for the cross-polarized (left panel) and collinear-polarized (right panel) pump and idler beams in the bare MoS_2 (the black curve) and the hybrid MoS_2-plasmonic structure (the red curve).

4. The full spectra of different nonlinear optical processes

In the experiment (the pump wavelength $\lambda_1 \approx$ 800nm; the idler wavelength $\lambda_2 \approx$1040 nm), the SHG at ~400 nm and 520 nm, SFG at ~452 nm and FWM at ~650 nm are marked in **Figure S4**. The FWM is enhanced as expected due to the pump frequency matching the longitudinal plasmonic resonance (ω_{p_L}) (i.e., $\omega_1=\omega_{p\_L}$). Besides, SHG of the idler light ($2\omega_2$) at 520 nm is also enhanced due to that the incident idler frequency is close to the plasmon resonance. SHG of the pump light ($2\omega_1$) at ~400 nm and SFG ($\omega_1+\omega_2$) at ~452 nm, however, are slightly enhanced mainly because the enhanced signals are partially reabsorbed by the gold structure. Furthermore, as the conversion efficiency of these second and third-order nonlinear optical processes in the hybrid MoS_2-plasmon nanostructures is in the order of ~10^{-7}, the competition among different nonlinear optical processes could be negligible, which is also confirmed by the experiments.

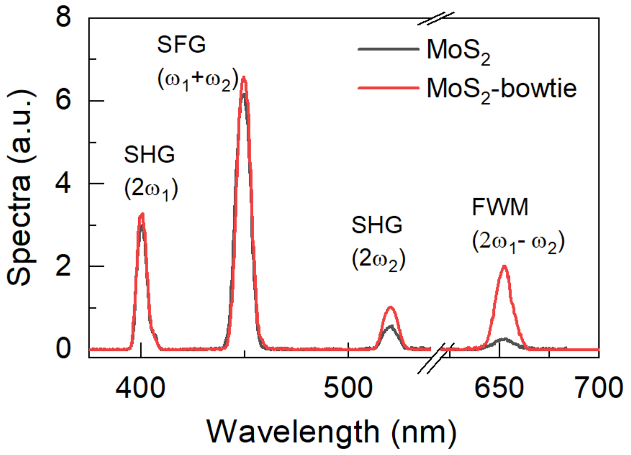

Figure S4 Generated full spectra with different nonlinear optical processes. SHG ($2\omega_1$, $2\omega_2$) at ~400 nm and 520 nm, SFG ($\omega_{SFG} = \omega_1 + \omega_2$) at ~452 nm, and FWM ($\omega_{FWM} = 2\omega_1 - \omega_2$) at ~650 nm in monolayer bare MoS_2 (black curve) and hybrid MoS_2-plasmonic structures (red curve) with excitation wavelengths λ_1 at ~800 nm and λ_2 at ~1040 nm.

5. Numerical simulation method

Three-dimensional finite-difference time-domain (FDTD) simulations are performed to understand the optical properties of the hybrid MoS_2-Au bowtie plasmonic structures on the SiO_2/Si substrate. The finest mesh size is set to be 0.1 nm in the structure. Perfectly matched layers are introduced in upper and lower boundaries in *z* direction and periodic boundary conditions are applied in *x* and *y* directions. The geometries of the metal nanostructures in the simulations are designed to match with the SEM images (Figure 1c in the main text). The refractive index of SiO_2 was set to n= 1.46. The refractive index of MoS_2, is obtained from Reference 3 and the thickness of MoS_2 is set to 0.7 nm. A vertically incident broadband planewave source is employed by using the total-field scattered-field technique.

The simulated reflectance spectra agree well with the measured data, as shown in **Figure S5**. We present the experimental (solid curves) and simulated (dotted curves) relative reflectance spectra ($R = R_{\mathrm{MoS_2-bowtie}}/R_{\mathrm{sub}}$), where $R_{\mathrm{MoS_2-bowtie}}$ is the reflection from the hybrid MoS_2-plasmonic structures and R_{sub} is the reflection from a bare SiO_2/Si substrate. In Figure S5a, the transverse plasmonic resonances (i.e., the peak in the reflectance spectra) are shown, respectively. In Figure S5b, the plasmonic resonances show blue shift with decreasing structure sizes in both the experimental and simulated reflectance spectra. Furthermore, the small peaks at around 620 and 660 nm corresponding to the B- and A-excitonic states of MoS_2 are also clearly observed both in experimental and simulated reflectance spectra.

Electric field enhancement, for the gold bowtie plasmonic nanocavities on SiO_2/Si substrates is calculated via a field monitor at the resonance wavelength at the wavelent800 nm) in the simulation. The simulated electric field intensity is defined as the ratio of the simulated $|E|^2$ of the nanostructure to the simulated $|E_0|^2$ on the bare SiO_2/Si substrate, as show in Figure 3b in the main text.

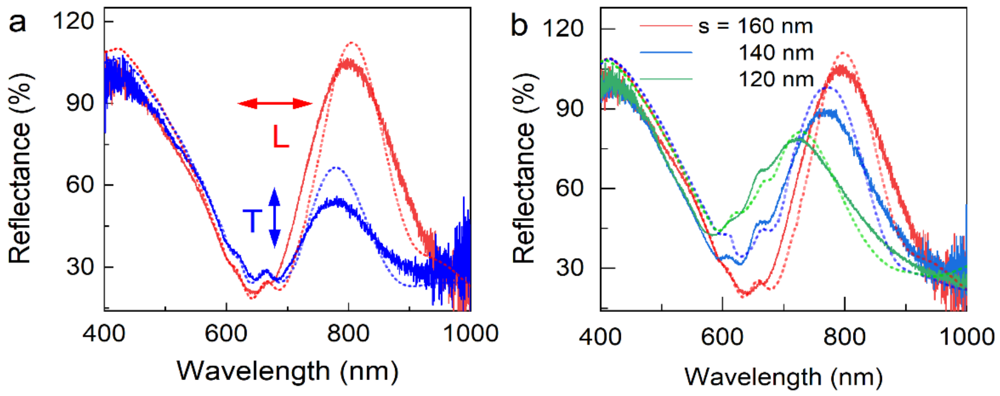

Figure S5 (a) The experimental and the simulated reflectance spectra of the hybrid MoS_2-plasmonic structures with different polarization directions. (b) The experimental and the simulated reflectance spectra of the hybrid MoS_2-plasmonic structures with different structure dimensions.

6. Polarization dependence of FWM enhancement

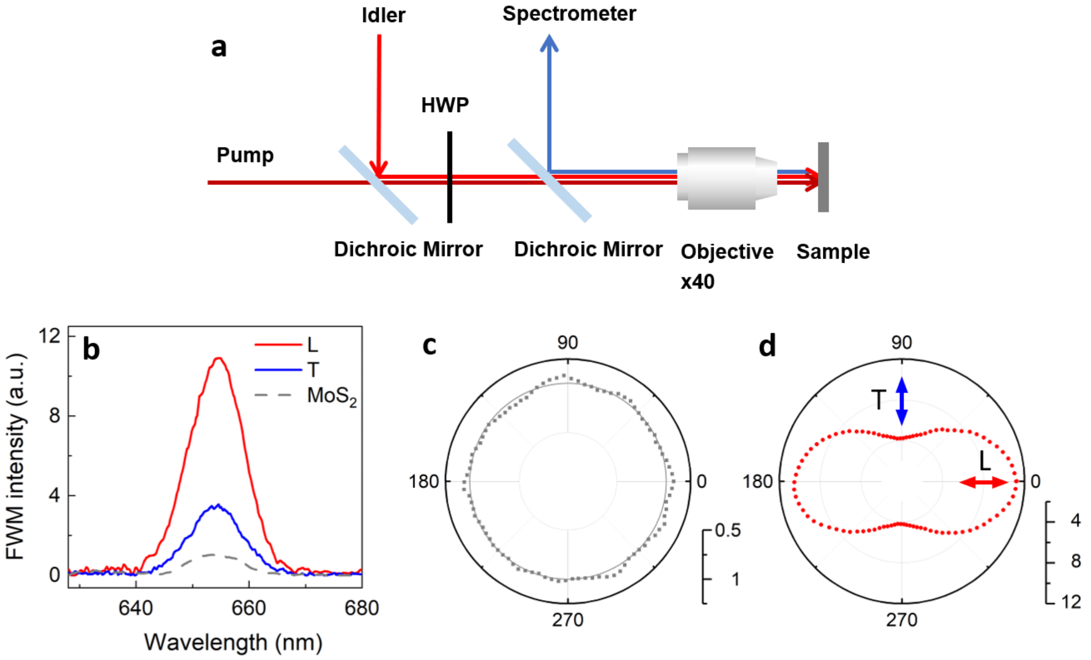

Figure S6 Angular dependence of the FWM in the hybrid MoS_2-plasmonic structure. (a) The illustration of the setup. The pump and idler beams are linearly polarized parallel with each other, and the polarization for both beams changes via the half waveplate (HWP). The angle between the polarization of incident beams and the *x*-axis is defined as α. (b) The enhanced FWM spectra of the MoS_2-plasmonic structure with the longitudinal (red curve) and transverse (blue curve) resonances of the plasmonic structures. The FWM from bare MoS_2 without plasmonic structures is also shown for a comparison (dashed gray curve). (c) The FWM intensity from bare MoS_2 is isotropic as a function of α. (d) The FWM intensity from the hybrid MoS_2-plasmonic structure as a function of α reaching the maximum at $\alpha = 0°$ (longitudinal plasmonic mode) and minimum at $\alpha = 90°$ (transverse plasmonic mode).

7. Theoretical calculation of the enhancement factors

For theoretically quantifying the plasmonic enhancement of the FWM process, the following calculations are conducted. When the pump and idler beams are normal incident on the monolayer MoS_2 with the polarizations parallel to each other, the nonlinear optical polarization $P^{(3)}$ of the FWM process is given by[4]

$$P^{(3)}(\omega_{\mathrm{FWM}}) = \chi^{(3)} E(\omega_1) E(\omega_1) E(\omega_2), \tag{1}$$

where $\omega_{\mathrm{FWM}} = 2\omega_1 - \omega_2$, $E(\omega_1)$ and $E(\omega_2)$ are the electric field of two incident beams. The intensity of FWM signals will be

$$I_{\mathrm{FWM}}(\omega_1, \omega_2) \propto P^{(3)}(\omega_{\mathrm{FWM}})^2 \propto |E(\omega_1)|^4 |E(\omega_2)|^2. \tag{2}$$

If the electric field is not uniform over the light-matter interaction region (e.g., manipulated by the nanostructures), the average FWM intensity in the region with area A will be

$$I_{\mathrm{FWM}}(\omega_1, \omega_2, A) \propto I(\omega_1, A) \cdot I(\omega_2, A)$$
$$= \frac{1}{A} \int_0^A |E(\omega_1, x, y)|^4 \, dxdy \cdot \frac{1}{A} \int_0^A |E(\omega_2, x, y)|^2 \, dxdy \tag{3}$$

where the electrical fields $E(\omega_1, x, y)$ and $E(\omega_2, x, y)$ can be obtained from theoretical simulation (see main text Figure 3b).

When the pump laser (λ_1 = ~800 nm) polarized along x-axis, the longitudinal plasmonic mode is excited in the MoS_2-plasmonic nanostructures. Thus, the electric field of the pump laser $E(\omega_1, x, y)$ is locally enhanced in the hot spot of the Au bowtie structure. As a result, $I_{\mathrm{th}}(\omega_{\mathrm{F}}, A_{\mathrm{gap}})$ in the hybrid MoS_2-plasmonic nanostructure increases accordingly. The excitation enhancement factor of the pump (f_{pump}) and idler (f_{idler}) beams can be determined as[5]

$$f_{pump}(\omega_1, A_{\mathrm{gap}}) = I(\omega_1, A_{\mathrm{gap}})_{\mathrm{MoS_2-bowtie}} / I(\omega_1)_{\mathrm{MoS_2}}. \tag{4}$$

$$f_{idler}(\omega_2, A_{\mathrm{gap}}) = I(\omega_2, A_{\mathrm{gap}})_{\mathrm{MoS_2-bowtie}} / I(\omega_2)_{\mathrm{MoS_2}}. \tag{5}$$

Then we consider the emission enhancement of the FWM signal in the hybrid MoS_2-plasmonic structures, the emission enhancement factor f_{em} of the FWM signal can be determined as[5]

$$f_{emi}(\omega_{\mathrm{FWM}}, A_{\mathrm{gap}}) = I(\omega_{\mathrm{FWM}}, A_{\mathrm{gap}})_{\mathrm{MoS_2-bowtie}} / I(\omega_{\mathrm{FWM}})_{\mathrm{MoS_2}}, \tag{6}$$

where $I(\omega_{\mathrm{FWM}}, A_{\mathrm{gap}})_{\mathrm{MoS_2-bowtie}} \propto \frac{1}{A_{\mathrm{gap}}} \int_0^{A_{\mathrm{gap}}} |E(\omega_{\mathrm{FWM}}, x, y)|^2 dxdy.$

Thus, the excitation enhancement factor EF_{th} can be determined as[5]

$$EF_{\mathrm{th}}(\omega_{\mathrm{FWM}}, A_{\mathrm{gap}}) = f_{pump}(\omega_1, A_{\mathrm{gap}}) \cdot f_{idler}(\omega_2, A_{\mathrm{gap}}) \cdot f_{em}(\omega_{\mathrm{FWM}}, A_{\mathrm{gap}}) \tag{7}$$

Here, we investigate the theoretical enhancement factor EF_{th} shown in Figure 5c of the manuscript. The theoretical enhancement factor EF_{th} includes the contribution of the pump and idler light, as well as the emission of FWM signals. As discussed above in Equation 7, the total

theoretical enhancement factor EF_{th} could be calculated as $EF_{th} = f_{pump} \cdot f_{idler} \cdot f_{emi}$, where f_{pump}, f_{idler} and f_{emi} are the enhancement factors of the pump, idler and emission of FWM signals, respectively. **Figure S7b** displays f_{emi} of the FWM emission at different FWM wavelengths (~588 – 734 nm) and f_{idler} of the idler light at the corresponding wavelengths (880 – 1260 nm) when the pump is fixed at ~800 nm. It is obvious that EF_{th} increases monotonously when both the idler and FWM wavelengths are approaching the plasmonic resonance (800 nm). And the local peaks (around 620 nm and 660 nm) of EF_{th} are attributed to the enhanced coupling between MoS_2 and plasmonic structure at exciton wavelengths for increased FWM emission.

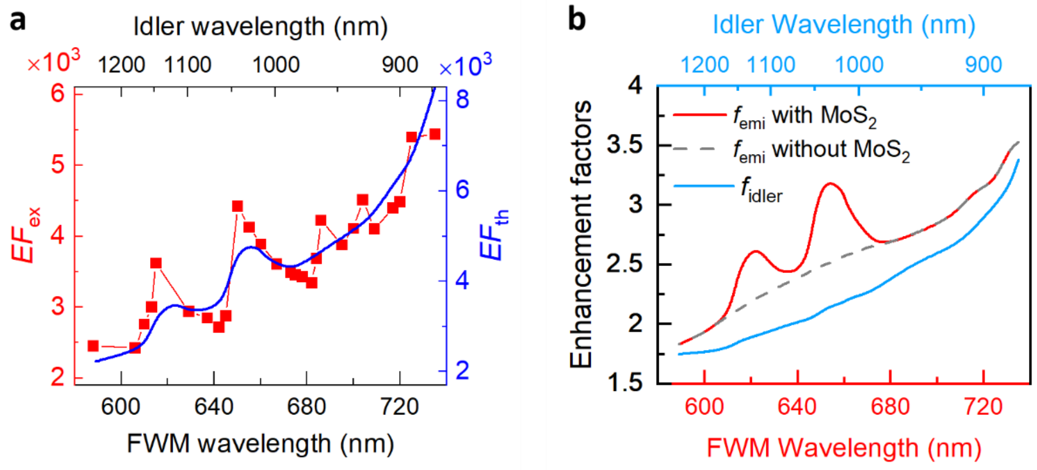

Figure S7 (a) Wavelength-dependent experimental (EF_{ex}, red dots) and theoretical (EF_{th}, blue curve) enhancement factors (i.e., Figure 5c of the manuscript). (b) The idler (f_{idler}) and emission (f_{emi}) enhancement factors increase when they approaches the plasmon resonance (i.e., 800 nm).

8. Calculation of nonlinear coefficients for FWM signals in MoS_2

The third-order nonlinear optical polarization $P^{(3)}$ induced in MoS_2 monolayer from FWM processes are given by[4]

$$P^{(3)}(\omega_F = 2\omega_1 - \omega_2) = \chi_s^{(3)} E(\omega_1)E(\omega_1)E(\omega_2),$$

where $E(\omega_1)$ and $E(\omega_2)$ are the electrical field of incident pump and idler incident light, $\chi_s^{(3)}$ is the nonlinear optical coefficients of monolayer MoS_2 sheet.

The intensity of FWM signal will be

$$I_{FWM} \propto |E(\omega_1)|^4 |E(\omega_2)|^2 \propto P_{ave}(\omega_1)^2 P_{ave}(\omega_2),$$

where $P_{ave}(\omega_i)$ is the average power of the incident light. This gives the law of power dependence in FWM process.

The output fields generated by the polarization $P^{(3)}$ is[4]

$$E(\omega_{\mathrm{F}}) = \alpha_{\mathrm{F}} \frac{1}{4} \frac{i\omega_{\mathrm{F}}}{2n_{\mathrm{F}}c} p^{(3)}(\omega_{\mathrm{F}}),$$

where $\alpha_{\mathrm{F}} = 3$ for two-color FWM.

The relation between the field and the corresponding average power is[4]

$$P_{ave}(\omega_i) = \frac{1}{8}\left(\frac{\pi}{\ln 2}\right)^{\frac{3}{2}} f\tau_i D_i^2 n_i \varepsilon_0 c \frac{|E(\omega_i)|^2}{2},$$

assuming pulses with repetition rate *f*, pulse duration τ_i, and a Gaussian spatial profile of diameter D_i on the sample, and n_i is the refractive index of the material. Here, *i* = 1, 2, F standing for pump, idler or generated FWM signals.

Therefore, the nonlinear optical coefficients of monolayer MoS_2 sheet can be calculated by

$$\left|\chi_{\mathrm{s}}^{(3)}(\omega_1, \omega_2; \omega_F)\right| = \frac{8n_{\mathrm{F}}c}{\alpha_{\mathrm{F}}\omega_{\mathrm{F}}} \frac{\varphi f \tau_1 D_1{}^2 n_1 \varepsilon_0 c}{P_{ave}(\omega_1)} \sqrt{\frac{\tau_2 D_2{}^2 n_2 P_{ave}(\omega_{\mathrm{F}})}{\tau_{\mathrm{F}} D_F{}^2 n_{\mathrm{F}} P_{ave}(\omega_2)}}.$$

The effective bulk-like third-order nonlinear coefficients of monolayer MoS_2 can be obtained, $\left|\chi^{(3)}(\omega_1, \omega_2; \omega_F)\right| = \left|\chi_{\mathrm{s}}^{(3)}(\omega_1, \omega_2; \omega_F)\right|/t$, where *t* = ~ 0.7 nm is the thickness of monolayer MoS_2.

In our experiment, the typical average power of pump ($P_{\mathrm{ave}}(\omega_1)$) and idler light ($P_{\mathrm{ave}}(\omega_2)$) is ~1 μW for each light beam. And *f* = 2 kHz is the repetition rate, and *τ*(1,2,F) = ~230 fs is the estimated pulse duration of pump light beams and FWM signals, and *D*(1,2,F) = ~2.5 μm is the diameter of the Gaussian spatial profile on the sample, and *n*(1,2,F) is refractive index of monolayer MoS_2 at the pump light frequencies and FWM frequencies[3], α = 3 for two-color FWM process, and *φ* = (π/ln2)3/2/8. For example, when $\hbar\omega_1$= 1.55 eV (i.e., λ_1 = 800 nm), $\hbar\omega_1$= 1.19 eV (i.e., λ_2 = 1040 nm), $\hbar\omega_1$= 1.91 eV (i.e., λ_{F} = 650 nm), the FWM signal from bare MoS_2 without plasmonic structures is measured as $P_{\mathrm{ave}}(\omega_F)$ = ~ 0.03 pW. Therefore, we obtain the bulk-like third-order nonlinear coefficient of the monolayer MoS_2 $|\chi^{(3)}| \approx 5 \times 10^{-18}$ V^2/m^2.

The equivalent $|\chi^{(3)}|$from the hybrid MoS_2-plasmonic structures can be calculated as,

$$\left|\chi^{(3)}(\omega_1, \omega_2; \omega_F)\right|_{\mathrm{eq}} = \sqrt{\frac{I_{\mathrm{MoS_2-bowtie}}}{I_{\mathrm{MoS_2}}}} \left|\chi^{(3)}(\omega_1, \omega_2; \omega_F)\right|$$

The calculated third-order equivalent nonlinear coefficient $\left|\chi^{(3)}(\omega_1, \omega_2; \omega_F)\right|_{\mathrm{eq}}$ of FWM in hybrid MoS_2-plasmonic structure and third-order nonlinear coefficient $\left|\chi^{(3)}(\omega_1, \omega_2; \omega_F)\right|$of bare MoS_2 are shown in Figure 5d in the main text.

9. Table S1 Typical $|\chi^{(3)}|$ reported on two-dimensional materials and conventional nonlinear materials, and $|\chi^{(3)}|$ of MoS_2 and hybrid MoS_2-plasmonic structures obtained from this work.

Nonlinear optical process	Material	$\|\chi^{(3)}\|$ (m^2/V^2)	References
FWM	MoS_2	~10^{-18} (bare MoS_2)<br>~10^{-17} (with plasmon)	**This work**
		10^{-18}	6
	Graphene	10^{-19}~10^{-17}	7
		10^{-15}	8
THG	MoS_2	10^{-19}~10^{-17}	9
		10^{-19}	10
		10^{-19}	11
	Graphene	10^{-19}~10^{-18}	7
	Gold thin film	10^{-19}	**This work,** 12, 13
	Si	10^{-18}	4
	TiO_2	10^{-20}	4
	BBO	10^{-22}~10^{-23}	14, 15
Z-Scan	$LiNbO_3$	10^{-21}	16, 17
	BBO	10^{-22}	16

10. Table S2 Enhancement factors for nonlinear coefficients of two-dimensional materials in hybrid structures.

Nonlinear optical process	Hybrid structures	$\|\chi^{(2)}\|$ (m/V) or $\|\chi^{(3)}\|$ (m^2/V^2) or enhancement factor (f)	References
FWM	MoS_2-Plasmon	$\|\chi^{(3)}\|$ ~10^{-17} m^2/V^2 (with plasmon)<br>f ~ 5000	**This work**
SHG	MoS_2-Plasmon	$\|\chi^{(2)}\|$ ~10^{-8} m/V(with plasmon)<br>f ~ 7000	18
	WS_2-Plasmon	f ~ 400	19
	WS_2-Photonic Crystal	f ~ 1000	20
	MoS_2-DBR	f ~ 60	21
THG	Graphene-Cavity	$\|\chi^{(3)}\|$ ~10^{-17} m^2/V^2 (in cavity)	22
	Nanowire-Cavity	f ~ 4.5	23

References

1. Li, Y.; Li, X.; Yu, T.; Yang, G.; Chen, H.; Zhang, C.; Feng, Q.; Ma, J.; Liu, W.; Xu, H.; Liu, Y.; Liu, X., Accurate Identification of Layer Number for Few-Layer WS_2 and WSe_2 via Spectroscopic Study. *Nanotechnol.* **2018,** *29* (12), 124001.

2. Dhakal, K. P.; Duong, D. L.; Lee, J.; Nam, H.; Kim, M.; Kan, M.; Lee, Y. H.; Kim, J., Confocal Absorption Spectral Imaging of MoS_2: Optical Transitions Depending on the Atomic Thickness of Intrinsic and Chemically Doped MoS_2. *Nanoscale* **2014,** *6* (21), 13028-35.

3. Hsu, C.; Frisenda, R.; Schmidt, R.; Arora, A.; Vasconcellos, S. M.; Bratschitsch, R.; Zant, H. S. J.; Castellanos-Gomez, A., Thickness-Dependent Refractive Index of 1L, 2L, and 3L MoS_2, $MoSe_2$, WS_2, and WSe_2. *Adv. Opt. Mater.* **2019,** *7* (13).

4. Boyd, R. W. *Nonlinear Optics*, 3rd Edition; Academic Press: New York, **2008**.

5. Lee, J.; Tymchenko, M.; Argyropoulos, C.; Chen, P. Y.; Lu, F.; Demmerle, F.; Boehm, G.; Amann, M. C.; Alu, A.; Belkin, M. A., Giant Nonlinear Response from Plasmonic Metasurfaces Coupled to Intersubband Transitions. *Nature* **2014,** *511* (7507), 65-9.

6. Dai, Y.; Wang, Y.; Das, S.; Xue, H.; Bai, X.; Hulkko, E.; Zhang, G.; Yang, X.; Dai, Q.; Sun, Z., Electrical Control of Interband Resonant Nonlinear Optics in Monolayer MoS_2. *ACS Nano* **2020,** *14* (7), 8442–8448.

7. Jiang, T.; Huang, D.; Cheng, J.; Fan, X.; Zhang, Z.; Shan, Y.; Yi, Y.; Dai, Y.; Shi, L.; Liu, K.; Zeng, C.; Zi, J.; Sipe, J. E.; Shen, Y.-R.; Liu, W.-T.; Wu, S., Gate-Tunable Third-Order Nonlinear Optical Response of Massless Dirac Fermions in Graphene. *Nat. Photon.* **2018,** *12* (7), 430-436.

8. Hendry, E.; Hale, P. J.; Moger, J.; Savchenko, A. K.; Mikhailov, S. A., Coherent nonlinear optical response of graphene. *Phys. Rev. Lett.* **2010,** *105* (9), 097401.

9. Saynatjoki, A.; Karvonen, L.; Rostami, H.; Autere, A.; Mehravar, S.; Lombardo, A.; Norwood, R. A.; Hasan, T.; Peyghambarian, N.; Lipsanen, H.; Kieu, K.; Ferrari, A. C.; Polini, M.; Sun, Z., Ultra-Strong Nonlinear Optical Processes and Trigonal Warping in MoS_2 Layers. *Nat. Commun.* **2017,** *8* (1), 893.

10. Woodward, R. I.; Murray, R. T.; Phelan, C. F.; Oliveira, R. E. P. d.; Runcorn, T. H.; Kelleher, E. J. R.; Li, S.; Oliveira, E. C. d.; Fechine, G. J. M.; Eda, G.; Matos, C. J. S. d., Characterization of the Second- and Third-Order Nonlinear Optical Susceptibilities of Monolayer MoS_2 Using Multiphoton Microscopy. *2D Materials* **2017,** *4* (1), 011006.

11. Autere, A.; Jussila, H.; Marini, A.; Saavedra, J. R. M.; Dai, Y.; Säynätjoki, A.; Karvonen, L.; Yang, H.; Amirsolaimani, B.; Norwood, R. A.; Peyghambarian, N.; Lipsanen, H.; Kieu, K.; de Abajo, F. J. G.; Sun, Z., Optical Harmonic Generation in Monolayer Group-VI Transition Metal Dichalcogenides. *Phys. Rev. B* **2018,** *98* (11), 115426.

12. Renger, J.; Quidant, R.; van Hulst, N.; Novotny, L., Surface-Enhanced Nonlinear Four-Wave Mixing. *Phys. Rev. Lett.* **2010,** *104* (4), 046803.

13. Burns, W. K.; Bloembergen, N., Third-Harmonic Generation in Absorbing Media of Cubic or Isotropic Symmetry. *Phys. Rev. B* **1971,** *4* (10), 3437-3450.
14. Banks, P. S.; Feit, M. D.; D., P. M., High-Intensity Third-Harmonic Generation. *J. Opt. Soc. Am. B* **2002,** *19*, 010102.
15. Tomov, I. V.; Van Wonterghem, B.; M., R. P., Third-Harmonic Generation in Barium Borate. *Appl. Opt.* **1992,** *31*, 4172-4174.
16. Ganeev, R. A.; Kulagin, I. A.; Ryasnyansky, A. I.; Tugushev, R. I.; Usmanov, T., Characterization of Nonlinear Optical Parameters of KDP, $LiNbO_3$ and BBO Crystals. *Opt. Commun.* **2004,** *229* (1-6), 403-412.
17. Kulagin, I. A.; Ganeev, R. A.; Tugushev, R. I.; Ryasnyansky, A. I.; Usmanov, T., Components of the Third-Order Nonlinear Susceptibility Tensors in KDP, DKDP and $LiNbO_3$ Nonlinear Optical Crystals. *Quantum Electron.* **2004,** *34* (7), 657-662.
18. Wang, Z.; Dong, Z.; Zhu, H.; Jin, L.; Chiu, M. H.; Li, L. J.; Xu, Q. H.; Eda, G.; Maier, S. A.; Wee, A. T. S.; Qiu, C. W.; Yang, J. K. W., Selectively Plasmon-Enhanced Second-Harmonic Generation from Monolayer Tungsten Diselenide on Flexible Substrates. *ACS Nano* **2018,** *12* (2), 1859-1867.
19. Shi, J.; Liang, W.-Y.; Raja, S. S.; Sang, Y.; Zhang, X.-Q.; Chen, C.-A.; Wang, Y.; Yang, X.; Lee, Y.-H.; Ahn, H.; Gwo, S., Plasmonic Enhancement and Manipulation of Optical Nonlinearity in Monolayer Tungsten Disulfide. *Laser Photon. Rev.* **2018,** *12* (10), 1800188.
20. Guo, Q.; Ou, Z.; Tang, J.; Zhang, J.; Lu, F.; Wu, K.; Zhang, D.; Zhang, S.; Xu, H., Efficient Frequency Mixing of Guided Surface Waves by Atomically Thin Nonlinear Crystals. *Nano Lett.* **2020,** *20* (11), 7956-7963.
21. Chen, Y. C.; Yeh, H.; Lee, C. J.; Chang, W. H., Distributed Bragg Reflectors as Broadband and Large-Area Platforms for Light-Coupling Enhancement in 2D Transition-Metal Dichalcogenides. *ACS Appl. Mater. Interfaces* **2018,** *10* (19), 16874-16880.
22. Beckerleg, C.; Constant, T. J.; Zeimpekis, I.; Hornett, S. M.; Craig, C.; Hewak, D. W.; Hendry, E., Cavity Enhanced Third Harmonic Generation in Graphene. *Appl. Phys. Lett.* **2018,** *112* (1), 011102.
23. Okhlopkov, K. I.; Shafirin, P. A.; Ezhov, A. A.; Orlikovsky, N. A.; Shcherbakov, M. R.; Fedyanin, A. A., Optical Coupling between Resonant Dielectric Nanoparticles and Dielectric Nanowires Probed by Third Harmonic Generation Microscopy. *ACS Photonics* **2019,** *6* (1), 189-195.
